# Physical activities and risk of neurodegenerative diseases: A two-sample Mendelian randomization study

**DOI:** 10.3389/fnagi.2022.991140

**Published:** 2022-09-23

**Authors:** Qiao Liao, Jian He, Kun Huang

**Affiliations:** ^1^Department of Neurology, Xiangya Hospital, Central South University, Changsha, China; ^2^National Clinical Research Center for Geriatric Disorders, Xiangya Hospital, Central South University, Changsha, China; ^3^Department of Gastroenterology, Nanfang Hospital, Southern Medical University, Guangzhou, Guangdong, China

**Keywords:** physical activity, neurodegenerative diseases, amyotrophic lateral sclerosis, Parkinson’s disease, Alzheimer’s disease, Mendelian randomization

## Abstract

**Objectives:**

Physical activity (PA) is considered beneficial in slowing the progression and improving the neurodegenerative disease prognosis. However, the association between PA and neurodegenerative diseases remains unknown. In this study, we conducted a two-sample Mendelian randomization (MR) analysis to estimate the causal association between PA phenotypes and neurodegenerative diseases.

**Materials and methods:**

Genetic variants robustly associated with PA phenotypes, used as instrumental variables, were extracted from public genome-wide association study (GWAS) summary statistics. Neurodegenerative diseases, including amyotrophic lateral sclerosis (ALS), Parkinson’s disease (PD), and Alzheimer’s disease (AD), were considered outcomes. GWAS information was also obtained from the most recent large population study of individuals with European ancestry. Multiple MR methods, pleiotropy tests and sensitivity analyses were performed to obtain a robust and valid estimation.

**Results:**

We found a positive association between moderate-to-vigorous physical activities and ALS based on the inverse variance weighted MR analysis method (OR: 2.507, 95% CI: 1.218–5.160, *p* = 0.013). The pleiotropy test and sensitivity analysis confirmed the robustness and validity of these MR results. No causal effects of PA phenotypes were found on PD and AD.

**Conclusion:**

Our study indicates a causal effect of PA on the risk of neurodegenerative diseases. Genetically predicted increases in self-reported moderate-to-vigorous PA participation could increase the risk of ALS in individuals of European ancestry. Precise and individualized prescriptions of physical activity should be provided to the elderly population.

## Introduction

With the aging of the population, the incidence of neurodegenerative diseases is increasing. Neuronal deterioration and degeneration lead to a plethora of clinical neurological deficits, including motor dysfunction and cognitive decline (Kiernan et al., 2011; Knopman et al., 2021). The most common age-related neurodegenerative diseases include amyotrophic lateral sclerosis (ALS), Parkinson’s disease (PD), and Alzheimer’s disease (AD), all of which exert a vast burden on individual families and society as a whole (Macpherson et al., 2017). To date, there are no curative therapies for neurodegenerative diseases. However, some modifiable factors, including physical activity (PA) and dietary habits, have been considered beneficial in slowing disease progression and improving disease prognosis (Grazioli et al., 2017; Mak et al., 2017; Gubert et al., 2020). Targeting these modifiable factors could also be effective in alleviating symptoms and relieving the abovementioned burdens.

Physical activity has recently attracted neurologists’ attention due to the mounting evidence supporting its role in maintaining brain health (Stillman and Erickson, 2018; Di Liegro et al., 2019; Donofry et al., 2021). Moderate PA has been related to the regulation of neuroplasticity and cell death from molecular perspectives and cognitive and motor functions at the behavioral level (de Sousa Fernandes et al., 2020; Liang et al., 2021; Maugeri et al., 2021). PA can promote autophagy and modulate mitophagy (Almeida et al., 2018), which play an important role in the pathogenesis of neurodegenerative diseases. Therefore, PA is thought to be beneficial for individuals experiencing neurodegenerative diseases, and its protective and preventive effects have been repeatedly noticed (Karssemeijer et al., 2017; Jia et al., 2019; Fan et al., 2020; Mahalakshmi et al., 2020; Kouloutbani et al., 2022). Furthermore, it has been accepted that PA could affect the risk of developing neurodegenerative diseases (Hamer and Chida, 2009; Lian et al., 2019). However, most of the epidemiological findings were obtained from a single center or a small study population (Veldink et al., 2005; Feddermann-Demont et al., 2017). The findings are also not consistent, and thus the association between PA and neurodegenerative diseases remains controversial (Pupillo et al., 2014; Feddermann-Demont et al., 2017; Kivimaki et al., 2019). Hence, large-scale or multicenter investigations focusing on the relationship between PA and neurodegenerative diseases are still required to understand the therapeutic potential of PA.

Mendelian randomization (MR) is a burgeoning analytical approach for exploring the causal relationship between exposures and outcomes. Genetic variants robustly associated with the level of exposure are often used as instrumental variables (IVs) in MR to estimate these causative associations. Unlike traditional randomized controlled trials, MR can identify potential causative factors of diseases, provide more information on whether a particular factor is a cause or a result of a disease, and ascertain whether modifying a specific factor will provide benefits. MR has been widely used in neurological diseases and has discriminated many of their causative factors.

Therefore, we utilized the MR method to investigate and estimate the causal relationship between PA and neurodegenerative diseases in individuals with European ancestry and hypothesize that PA could reduce the risk of neurodegenerative diseases. In our study, a two-sample MR analysis was performed to detect the potential causal association between PA and the risk of neurodegenerative diseases (ALS, PD, and AD) by using genetic variants robustly associated with PA phenotypes, including self-reported and objective accelerometer-based PA levels obtained from published genome-wide association studies (GWASs).

## Materials and methods

### Genome-wide association study summary statistics for physical activities

Summary statistics for physical activities were obtained from a recently published GWAS of over 377,000 UK biobank participants (Klimentidis et al., 2018). This study identified significant genetic variants associated with PA engagement, which included self-reported and accelerometry-based levels of PA in daily and leisure-time exercise habits. For self-reported PAs, responses to a touchscreen questionnaire similar to the International Physical Activity Questionnaire (Craig et al., 2003) were collected and divided into two phenotypes, listed as self-reported moderate-to-vigorous physical activities (MVPA) and self-reported vigorous physical activities (VPA). For the accelerometry-based PAs, exercise data were recorded from a wrist-worn Axivity-AX3 accelerometer (Doherty et al., 2017). Two measurements derived from the portable device were examined, including the overall acceleration average (OAA) and fraction of accelerations > 425 milligravities (FAA); this cutoff value was determined in accordance with the metabolic equivalents of vigorous physical activities (Hildebrand et al., 2014; Klimentidis et al., 2018).

### Genome-wide association study summary statistics for neurodegenerative diseases

The genome-wide association data for ALS were obtained from publicly available GWAS summary statistics, including 20,806 ALS patients and 59,804 control individuals of European ancestry (mainly from the United States, Italian, French, and Belgian) (Nicolas et al., 2018). Both familial and sporadic ALS were included in the GWAS analysis. All patients were diagnosed according to the El Escorial criteria, and all had an onset age older than 18 years. The most recent and largest GWAS of PD involving 482,730 European participants, conducted by the International Parkinson’s Disease Genomics Consortium, was used for the summary statistics for PD outcome. The study detected and reported more than 15 million SNPs in 33,674 PD patients and 449,056 controls (Nalls et al., 2019). We obtained GWAS summary data for AD from a GWAS meta-analysis in a total European population of 63,926 participants (21,982 AD patients and 41,944 cognitively normal controls) performed by the International Genomics of Alzheimer’s Project (Kunkle et al., 2019).

### Instrumental variable selection

To screen out significant single-nucleotide polymorphisms (SNPs) associated with PA as valid IVs, a cutoff *p*-value of 5 × 10^–8^ with genome-wide significance was chosen. In addition, the threshold of *r*^2^ was set as 0.001 to minimize the confounding factors. Consequently, in the PA GWAS, nine genome-wide significant SNPs for MVPA (*n* = 377,234) and five significant SNPs for VPA (*n* = 261,055) were separately identified. Furthermore, we identified eight and two SNPs with genome-wide significance for OAA and FAA, respectively. Considering that there were only 2 genome-wide significant SNPs for FAA, a more relaxed cutoff the *p*-value of 5 × 10^–7^ was used, and eight SNPs were consequently selected (Choi et al., 2019; Zhuo et al., 2021). In addition, the threshold for minor allele frequency was set to 0.01. When no SNPs of the PA phenotypes were found in the outcome GWAS, they were replaced by their proxies, which were identified from LDlink.^1^ Furthermore, we removed SNPs associated with confounders and outcomes based on the PhenoScanner V2 database.^2^ As listed in Supplementary Table 1, SNPs that fulfilled the three core requirements were finally selected as IVs to assess the causal relationship between PA and neurodegenerative diseases.

### Statistical analysis

**FIGURE 1:**
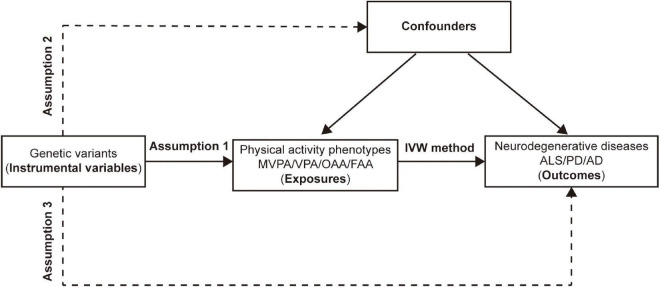
Assumptions and design of the two-sample mendelian randomization in this study. Genetic variants significantly associated with physical activity phenotypes are used as instrumental variables. MVPA, Self-reported moderate-to-vigorous physical activity; VPA, Self-reported vigorous physical activity; OAA, Overall acceleration average; FAA, Fraction of accelerations > 425 milli-gravities; IVW, Inverse variance weighted; ALS, amyotrophic lateral sclerosis; PD, Parkinson’s disease; AD, Alzheimer’s disease.

Mendelian randomization has been widely used to explore the causal effect of exposure on outcome (Liao et al., 2022a). Genetic variants that could explain the actual level of exposure are often used as IVs. In MR analysis, as shown in Figure 1, three fundamental assumptions should be satisfied to ensure the validity of the IVs: (1) the chosen IVs should be significantly associated with exposure (PA); (2) the available IVs should not be associated with any confounding factors of the exposure-outcome relationship; and (3) the chosen IVs should not affect the outcome, except possibly *via* the exposure-outcome relationship. In detail, genetic variants robustly associated with the PA phenotypes (namely, the exposure) were used as IVs, and neurodegenerative diseases, including ALS, PD, and AD, were regarded as outcomes. Thus, a two-sample MR was adopted as the main statistical approach to investigate the causal associations between each IV-exposure and IV-outcome (ALS, PD, AD) pairing.

The inverse variance weighted (IVW) method has been widely used in MR analysis, developed as a meta-analysis of the variant-specific causal estimates and used to obtain a pooled estimation of the causal effect. The IVW method has certain advantages over other similar methods, especially when the IVs enrolled in the analysis were robustly valid. When there were weak IVs, the MR–Egger method could also be applied to obtain a less biased causal estimation. In addition, to minimize the influential impact of abnormal SNPs, the median-based method was performed as a supplement to the IVW method. Finally, the maximum likelihood method was utilized when the populations overlapped.

After MR analysis, sensitivity analysis, including heterogeneity and pleiotropy, was performed. Cochran’s *Q* statistic was calculated to measure heterogeneity which is widely used (Huang et al., 2021; Liao et al., 2022b). A large *Q* statistic value indicates that the effect of individual PA phenotype-associated SNPs on outcomes enjoys a relatively large difference, which suggests the existence of heterogeneity and that the findings should be cautiously interpreted. Moreover, a random-effect IVW method was implemented to assess the causal association between the PA phenotypes and outcomes. To explore the directional pleiotropy of the PA phenotype-associated SNPs, the MR–Egger regression test was adopted by comparing the deviation of the MR–Egger intercept from zero, and an intercept *p*-value of less than 0.05 suggested the presence of horizontal pleiotropy. Furthermore, Mendelian Randomization Pleiotropy RESidual Sum and Outlier (MR-PRESSO) analysis was also performed to identify any possible horizontal pleiotropy (Verbanck et al., 2018). Additionally, leave-one-out analysis was used to determine the potential PA phenotype-associated SNPs that might influence the causal effect and identify potential outliers.

R software (version 4.1.1, for Windows) was used to conduct our two-sample MR. We adopted three main packages for statistical analysis, data output and visualization: (1) ‘‘TwoSampleMR,’’^3^ (2) ‘‘MRPRESSO,’’^4^ and (3) ‘‘forestplot.’’^5^ A two-tailed *p*-value less than 0.05 was considered statistically significant.

## Results

The summarized statistics of all selected SNPs of different PA phenotypes are listed in Supplementary Table 1. Based on the PhenoScanner V2 database, one of the nine MVPA related SNPs was associated with AD, so we omitted this SNP from the MR analysis of the MVPA-AD association.

### Physical activity with amyotrophic lateral sclerosis

**FIGURE 2:**
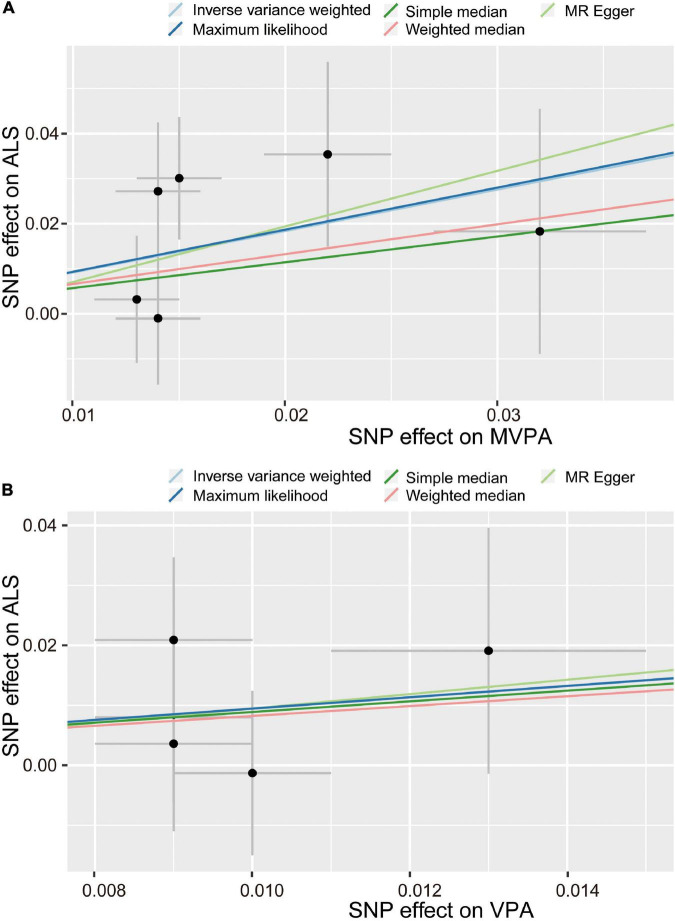
Scatter plot of causal effect of MVPA **(A)** and VPA **(B)** on ALS. MVPA, Self-reported moderate-to-vigorous physical activity; VPA, Self-reported vigorous physical activity; SNP, single nucleotide polymorphism; ALS, amyotrophic lateral sclerosis.

**FIGURE 3:**
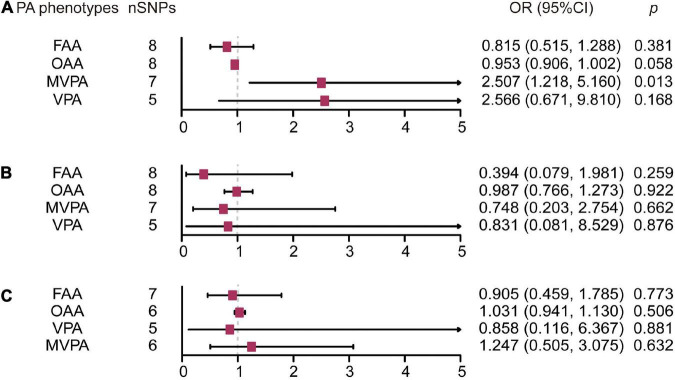
Forest plot of causal effect of physical activity phenotypes on amyotrophic lateral sclerosis **(A)**, Parkinson’s disease **(B)**, and Alzheimer’s disease **(C)** estimated by the inverse variance weighted method. PA, physical activity; SNP, single nucleotide polymorphism; MVPA, Self-reported moderate-to-vigorous physical activity; VPA, Self-reported vigorous physical activity; OAA, Overall acceleration average; FAA, Fraction of accelerations > 425 milli-gravities; OR, odds ratio; CI, confidence interval.

For the MVPA phenotype, no evidence of heterogeneity was present according to Cochran’s *Q* statistic (*Q* = 5.270, *p* = 0.384). As shown in Figures 2A, 3A, we found evidence of a positive association between MVPA and ALS based on the IVW method (OR: 2.507, 95% CI: 1.218–5.160, *p* = 0.013), indicating that a genetically determined increased MVPA engagement could increase the risk of ALS. The maximum likelihood method yielded a similar positive effect (OR: 1.669, 95% CI: 1.220–5.309, *p* = 0.013). In addition, the other MR results shared similar patterns of effect (Table 1), although the statistical significance was absent (MR–Egger OR: 3.433, 95% CI: 0.218–54.024, *p* = 0.421; simple median OR: 1.772, 95% CI: 0.631–4.974, *p* = 0.278; weighted median OR: 1.939, 95% CI: 0.709–5.300, *p* = 0.197). No directional pleiotropy was suggested by the MR–Egger intercept with an intercept value of −0.005 and *p*-value of 0.826 (Table 2). MR-PRESSO analysis also indicated that there was no horizontal pleiotropy (Table 2), so there were no outliers for any IVs (global test: *p* = 0.813). The leave-one-out test showed that the causal estimation of genetically predicted MVPA on ALS risk was mostly not influenced by a single SNP, implying the robustness and validity of our results. For the VPA phenotype, our results suggested a consistent trend of the causal estimation that VPA engagement could promote the onset of ALS across different statistical methods, although the *p*-values were not significant (Figure 2B and Table 1). No evidence of heterogeneity or pleiotropy was shown (shown in Table 2). For OAA, according to the maximum likelihood method, an inverse association between OAA and ALS risk was revealed (OR: 0.951, 95% CI: 0.907–0.997, *p* = 0.036). However, other MR methods yielded different trends (Table 2). For FAA, our MR analysis indicated no causal association between FAA and ALS risk by either the IVW method or other methods (shown in Table 1 and Figure 3A).

**TABLE 1:** Estimated association between physical activity phenotypes and amyotrophic lateral sclerosis.

PA phenotypes	MR methods	Number of SNPs	*P*	OR (95% CI)
MVPA	IVW	7	0.013*	2.507 (1.218–5.160)
	MR Egger	7	0.421	3.433 (0.218–54.024)
	Maximum likelihood	7	0.013*	2.545 (1.220–5.309)
	Simple median	7	0.278	1.772 (0.631–4.974)
	Weighted median	7	0.197	1.939 (0.709–5.300)
VPA	IVW	5	0.168	2.566 (0.671–9.810)
	MR Egger	5	0.840	3.318 (0–145568.627)
	Maximum likelihood	5	0.168	2.575 (0.670–9.896)
	Simple median	5	0.310	2.432 (0.437–13.528)
	Weighted median	5	0.339	2.277 (0.421–12.317)
OAA	IVW	8	0.058	0.953 (0.906–1.002)
	MR Egger	8	0.656	1.057 (0.839–1.331)
	Maximum likelihood	8	0.036*	0.951 (0.907–0.997)
	Simple median	8	0.068	0.947 (0.894–1.004)
	Weighted median	8	0.069	0.944 (0.887–1.004)
FAA	IVW	8	0.381	0.815 (0.515–1.288)
	MR Egger	8	0.257	0.013 (0.000–11.571)
	Maximum likelihood	8	0.336	0.809 (0.526–1.245)
	Simple median	8	0.868	1.050 (0.590–1.867)
	Weighted median	8	0.830	1.066 (0.598–1.900)

*p-value less than 0.05 is considered statistically significant.

PA, physical activity; MR, mendelian randomization; SNP, single nucleotide polymorphism; MVPA, Self-reported moderate-to-vigorous physical activity; VPA, Self-reported vigorous physical activity; OAA, Overall acceleration average; FAA, Fraction of accelerations > 425 milli-gravities; IVW, Inverse variance weighted; OR, odds ratio; CI, confidence interval.

**TABLE 2:** Sensitivity test of the mendelian randomization analysis between physical activity phenotypes and amyotrophic lateral sclerosis.

PA phenotypes	MR methods	Heterogeneity test		Horizontal pleiotropy		MR-PRESSO	Steiger test
					
		Cochran’s Q	*P*	Egger_intercept	*P*	*P* of global test	*P*
MVPA				−0.005	0.826	0.514	<0.001*
	IVW	5.327	0.503				
	MR-Egger	5.270	0.384				
VPA				−0.003	0.965	0.813	0*
	IVW	1.647	0.800				
	MR-Egger	1.645	0.649				
OAA				−0.028	0.400	0.312	0*
	IVW	8.436	0.296				
	MR-Egger	7.423	0.283				
FAA				0.103	0.277	0.354	0*
	IVW	8.188	0.316				
	MR-Egger	6.613	0.358				

*p-value less than 0.05 is considered statistically significant.

PA, physical activity; MR, mendelian randomization; MVPA, Self-reported moderate-to-vigorous physical activity; VPA, Self-reported vigorous physical activity; OAA, Overall acceleration average; FAA, Fraction of accelerations > 425 milli-gravities; IVW, Inverse variance weighted.

### Physical activity with Parkinson’s disease

For the self-reported PA phenotype, including MVPA and VPA, shown in Supplementary Table 4, there was no evidence of heterogeneity as measured by the Q statistics (MVPA *Q* = 8.211, *p* = 0.145; VPA *Q* = 6.558, *p* = 0.087). Multiple MR methods, including the IVW method, showed that the trend of the effect of MVPA on PD risk was consistent that the risk of PD tended to decrease when the genetically determined MVPA engagement increased despite the association was non-significant (Supplementary Table 2 and Figure 3B). Similar findings were also observed in the causal effect estimation between VPA and PD. MR–Egger intercept and MR-PRESSO analyses found no evidence for any pleiotropy in the MVPA-PD and VPA-PD associations, indicating the stability of our analysis (Supplementary Table 4). For accelerometry-based PA, heterogeneity was detected in both the OAA and FAA phenotypes, as indicated by the large Q statistics (OAA *Q* = 89.134, *p* = 0.000; FAA *Q* = 33.208, *p* < 0.001). Thus, a random-effects model for the IVW method was utilized to investigate the causal relation between these two phenotypes and PD (Supplementary Table 2). Neither OAA nor FAA exerted a causal effect on the possibility of developing PD (OAA OR: 0.987, 95% CI 0.766–1.273, *p* = 0.922; FAA OR: 0.394, 95% CI: 0.079–1.981, *p* = 0.259).

### Physical activity and Alzheimer’s disease

There was no evidence of heterogeneity in the MVPA-AD relationship (*Q* = 2.730, *p* = 0.604); thus, the IVW, MR–Egger, median-based and maximum likelihood methods were all used to evaluate the causation of MVPA on AD (Supplementary Table 4). Demonstrated in Supplementary Table 3 and Figure 3C, it was implied that there was no significant causal association, and the effect pattern varied for the different methods (IVW: OR: 1.247, 95% CI: 0.505–3.075, *p* = 0.632; MR Egger OR: 19.620, 95% CI: 0.96–401.027, *p* = 0.125; weighted median OR: 1.288, 95% CI: 0.430–3.853, *p* = 0.651; simple median OR: 0.995, 95% CI: 0.337–2.932; *p* = 0.992). As heterogeneity was detected in the VPA-AD, OAA-AD, and FAA-AD relationships, the random-effects IVW method was used to estimate whether these were causal relationships (Supplementary Table 3 and Figure 3C). The analysis showed VPA and FAA were both inversely associated with AD risk, although the association was not significant (VPA OR: 0.858, 95% CI: 0.116–6.367, *p* = 0.881; FAA OR: 0.905, 95% CI: 0.459–1.785, *p* = 0.773), while OAA was positively related to AD risk but also not significant (OAA OR: 1.031, 95% CI: 0.941–1.13, *p* = 0.506).

## Discussion

In this study, we conducted two-sample MR to investigate the causal relationship between PA and neurodegenerative diseases. Although it has been previously reported that PA could reduce the risk of developing neurodegenerative diseases, our results demonstrated that different physical phenotypes exert different effect on neurodegenerative diseases and there was only a causal association between MVPA and ALS. It was revealed that genetically determined increased MVPA engagement could significantly increase the risk of ALS.

Amyotrophic lateral sclerosis is a rare and fatal neurodegenerative disease caused by degeneration of the motor neurons (Shi et al., 2022). The effect of PA on ALS continues to be controversial. Some studies have revealed that PA is not a risk factor for ALS and that increased PA does not increase susceptibility to ALS (Valenti et al., 2005; Pupillo et al., 2014; Feddermann-Demont et al., 2017). However, these studies included limited populations, the conclusions were not validated. Recently, some studies with a larger population size have shown opposite results. A multicenter case–control study suggested that a higher risk of ALS is associated with PA in leisure time and occupational activities after adjustment for confounders (Visser et al., 2018). Furthermore, a cross-sectional study also indicated that patients who reported at least moderate engagement in PA (three times a week) during early adulthood were more likely to be diagnosed with ALS than those who did not (Raymond et al., 2021). Our analysis also supports that more involvement in PA is associated with ALS risk.

Among the selected methods in our analysis, with the validity of the IVs and the absence of pleiotropy, the IVW method was the most efficient (Zhu, 2021). Specifically, in the analysis of MVPA, the causal relationship was significant in two different analytical approaches including the IVW and the maximum likelihood, and the direction of effect remained similar. However, in the analysis of OAA, the significant result was only yielded by the maximum likelihood methods and the tendency were not consistent by different methods which weakened the validity and robustness of the results. Thus, our results demonstrated that more MVPA engagement was casually related with ALS risk. It needs intensive investigation whether PA could alter the risk of ALS by interfering with some already known ALS etiologies. It has been reported that different intensities of PA could induce various glucose demands which leads to the alteration of the muscle metabolism and autophagy flux, and provide different effect in ALS mice (Desseille et al., 2017). Besides, different intensities of PA could also the alter the sizes of recruited motoneurons and modulate the neurotrophic signal in the neuromuscular junctions (Just-Borras et al., 2020). Anyway, the underlying molecular mechanisms between different PA phenotypes and ALS risk still require further exploration. Uncovering the relationship between PA and ALS will be beneficial in the development of more precise guidance for ALS patients.

In addition, we explored the effect of PA on PD. In contrast to the effect of PA on ALS, our results showed that PA was not causally associated with PD risk but genetically predicted increased engagement in MVPA and VPA were inclined to protect against PD. Previous study has found that there is no obvious difference in PA levels between PD and healthy controls (Amara et al., 2019), which is inconsistent with our results. Besides, higher PA levels are associated with slower PD progression (Amara et al., 2019). Various studies have proposed that physical activity interventions could be effective in alleviating the motor and non-motor symptoms of PD, and in improving multiple outcomes in people with PD (Morberg et al., 2014; Okun, 2017; Cheng and Su, 2020; Hidalgo-Agudo et al., 2020). Our results did not invalidate these former studies despite no causality was found between PA and PD risk. Besides, a future investigation of the causal relationship between PA and PD progression are needed. On the other hand, it should be noted that PA could directly interfere with the PD-related pathophysiological process (Fan et al., 2020). In a PD rat model, early moderate treadmill running practice mitigates the accumulation of alpha-synuclein, which contributes to PD pathogenesis and motor dysfunction, and maintains levels of tyrosine hydroxylase in the substantia nigra, which plays an essential role in the metabolism of dopamine (Almeida et al., 2018). Additionally, it is suggested in PD mice model that PA could induce neuroprotection against cell death by promoting enhanced autophagy and neuronal regeneration (Jang et al., 2018), and by regulating both mitochondrial function and neuroinflammation (Tuon et al., 2015). Therefore, disclosing the role of PA in PD risk and progression would be of great benefit and could promote the prescription of PA as an efficient treatment method for PD.

It was previously reported that PA could improve cognitive function (Anderson-Hanley et al., 2012; Holthoff et al., 2015; Palta et al., 2019; Zhou et al., 2022), but the relationship between PA and AD risk remains unclear. Our analysis showed that there was no causal effect of either self-reported PA or accelerometry-based PA levels on AD onset, and the extent of engagement in PA was not a risk factor for AD. Our analysis is consistent with study results derived from meta-analysis of 19 prospective observational cohort studies. It contains more than 400,000 participants with a follow-up of at least 10 years demonstrated that less PA was not associated with a higher risk of dementia and AD (Kivimaki et al., 2019). Besides, existing evidence supporting that PA could reduce the risk of AD or dementia is insufficient (Brasure et al., 2018).

It is worth noting that the effect of PA on ALS, PD, and AD varies, which means that the roles of PA in neurodegenerative disease might differ from each other, and that there is no one-size-fits-all PA prescription for neurodegenerative diseases. It is necessary for clinicians to offer individualized and precise PA guidance for individuals with neurodegenerative disease.

There are some major strengths in our study. First, we explored for the first time the causal effect of different phenotypes of PA on neurodegenerative diseases by two-sample MR analysis. Second, we adopted multiple MR methods, including inverse variance weighted analysis, the MR–Egger method, the median-based method and the maximum likelihood method, to robustly estimate causal relationships, MR–Egger regression and MR-PRESSON to detect pleiotropy, and leave-one-out as the sensitivity test to examine the validity of the results. Third, the random-effect IVW method was implemented to assess the causal association if heterogeneity was present.

However, our MR analysis has some limitations. First, since the GWAS databases of PA and neurodegenerative diseases were all derived from individuals of European ancestry, the universality of our results is limited. Further studies are required to demonstrate whether our results are consistent for other ancestries. Second, although we ruled out SNPs associated with confounders in the MR analysis, some other unknown confounders are present that might influence the causal estimation. Third, PA engagement is determined not only by genetic factors but also by other factors; thus, it should be noted that our results could only explain the causal association of PA with neurodegenerative diseases. Fourth, the included SNPs significantly associated with PA are limited, which could decrease the statistical validity of our findings while minimizing the confounding influence and pleiotropy. Lastly, as the information of ALS, PD and AD phenotypes including genetic mutations are insufficient, the causality of PA on different phenotypes are unable to valuate. Therefore, further investigations to identify more genetic loci associated with PA engagement are needed.

## Conclusion

To our knowledge, our MR analysis is the first to indicate a causal effect of PA on the risk of neurodegenerative diseases. Genetically predicted increases in MVPA participation could increase the risk of ALS in individuals of European ancestry. Precise and individualized prescriptions of physical activity should be provided to the elder population.

## Data availability statement

The original contributions presented in this study are included in the article/Supplementary material, further inquiries can be directed to the corresponding author.

## Author contributions

KH conceived the project. QL and KH extracted data and performed the analysis. QL wrote the manuscript. All authors polished the manuscript and critically revised it for valuable intellectual content and approved the final manuscript.

## Footnotes

1
https://ldlink.nci.nih.gov/

2
http://www.phenoscanner.medschl.cam.ac.uk/

3
https://github.com/MRCIEU/TwoSampleMR

4
https://github.com/rondolab/MR-PRESSO

5
https://cran.r-project.org/web/packages/forestplot/index.html

## Abbreviations

ALS: amyotrophic lateral sclerosis
PD: Parkinson’s disease
AD: Alzheimer’s disease
PA: physical activity
MR: mendelian randomization
SNP: single nucleotide polymorphism
MVPA: self-reported moderate-to-vigorous physical activity
VPA: self-reported vigorous physical activity
OAA: overall acceleration average
FAA: fraction of accelerations > 425 milli-gravities
IVW: inverse variance weighted
OR: odds ratio
CI: confidence interval.
