# Supplementary material for: Physical activities and risk of neurodegenerative diseases: A two-sample Mendelian randomization study
Source: Front Aging Neurosci. 2022 Sep 23;14:991140. doi: 10.3389/fnagi.2022.991140 (PMC9541335; doi:10.3389/fnagi.2022.991140)
Supplement: Supplementary file 1 [file Table_1.DOCX]

Additional file 1: Summary characteristics of selected genetic variants associated with physical activity phenotypes used as instrumental variables.

| PA phenotypes | SNP | position | EA | OA | EAF | b | se | *p* | sample size |
| --- | --- | --- | --- | --- | --- | --- | --- | --- | --- |
| MVPA | rs3094622 | 6: 30327952 | A | G | 0.865 | 0.02 | 0.003 | 1.40E-09 | 377234 |
| MVPA | rs7804463 | 7: 133447651 | T | C | 0.53 | 0.015 | 0.002 | 1.20E-11 | 377234 |
| MVPA | rs7791992 | 7: 50237784 | C | A | 0.413 | -0.014 | 0.002 | 5.70E-10 | 377234 |
| MVPA | rs429358^*^ | 19: 45411941 | T | C | 0.846 | -0.022 | 0.003 | 6.10E-13 | 377234 |
| MVPA | rs2988004 | 9: 37044388 | T | G | 0.558 | -0.013 | 0.002 | 4.10E-09 | 377234 |
| MVPA | rs2854277 | 6: 32628084 | C | T | 0.917 | 0.032 | 0.005 | 2.60E-10 | 377234 |
| MVPA | rs2035562 | 3: 85056521 | A | G | 0.328 | -0.014 | 0.002 | 3.90E-09 | 377234 |
| MVPA | rs149943 | 6: 28002388 | G | A | 0.853 | 0.019 | 0.003 | 2.20E-09 | 377234 |
| MVPA | rs1043595 | 7: 128410012 | G | A | 0.717 | 0.014 | 0.002 | 4.30E-09 | 377234 |
| VPA | rs328902 | 7: 35020843 | C | T | 0.685 | -0.009 | 0.001 | 5.50E-10 | 261055 |
| VPA | rs3781411 | 10: 126715436 | C | T | 0.876 | 0.013 | 0.002 | 3.00E-10 | 261055 |
| VPA | rs13243553 | 7: 133506955 | G | A | 0.608 | 0.009 | 0.001 | 9.00E-11 | 261055 |
| VPA | rs2764261 | 6: 108927842 | A | G | 0.374 | 0.009 | 0.001 | 2.00E-11 | 261055 |
| VPA | rs1248860 | 3: 85015779 | G | A | 0.484 | -0.01 | 0.001 | 1.10E-13 | 261055 |
| OAA | rs59499656 | 18: 40768309 | A | T | 0.656 | -0.228 | 0.038 | 2.40E-09 | 91084 |
| OAA | rs55657917 | 17: 43844560 | T | G | 0.78 | -0.303 | 0.044 | 5.00E-12 | 91084 |
| OAA | rs148193266 | 11: 104528681 | A | C | 0.957 | -0.51 | 0.092 | 3.10E-08 | 91084 |
| OAA | rs11012732 | 10: 21830104 | A | G | 0.668 | 0.225 | 0.039 | 5.40E-09 | 91084 |
| OAA | rs9293503 | 5: 87948962 | T | C | 0.888 | 0.329 | 0.059 | 2.10E-08 | 91084 |
| OAA | rs12522261 | 5: 152054825 | G | A | 0.657 | 0.211 | 0.038 | 3.90E-08 | 91084 |
| OAA | rs6775319 | 3: 18758501 | A | T | 0.271 | 0.225 | 0.041 | 3.50E-08 | 91084 |
| OAA | rs34517439 | 1: 78450517 | C | A | 0.879 | 0.308 | 0.056 | 4.40E-08 | 91084 |
| FAA | rs1856329 | 1: 219939623 | A | C | 0.801 | 0.027 | 0.005 | 9.00E-08 | 90667 |
| FAA | rs1668835 | 18: 22478952 | T | A | 0.688 | -0.023 | 0.004 | 3.10E-07 | 90667 |
| FAA | rs80028338 | 17: 44161470 | A | C | 0.795 | -0.028 | 0.005 | 1.50E-07 | 90667 |
| FAA | rs743580 | 15: 74328116 | A | G | 0.51 | 0.025 | 0.004 | 1.30E-09 | 90667 |
| FAA | rs4754194 | 11: 107090187 | C | T | 0.773 | -0.025 | 0.005 | 2.40E-07 | 90667 |
| FAA | rs72633364 | 8: 34186888 | G | A | 0.711 | -0.023 | 0.005 | 4.10E-07 | 90667 |
| FAA | rs62443625 | 7: 39053131 | T | C | 0.767 | -0.026 | 0.005 | 1.40E-07 | 90667 |
| FAA | rs6433478 | 2: 175241482 | T | C | 0.457 | -0.024 | 0.004 | 1.20E-08 | 90667 |

^*^: The SNP associated with AD based on the PhenoScanner V2 database which are omitted in the MR analysis of MVPA-AD association.

Abbreviations: PA, physical activity; SNP, single nucleotide polymorphism; EA, effect allele; OA, other allele; EAF, effect allele frequency; se, standard error; MVPA, Self-reported moderate-to-vigorous physical activity; VPA, Self-reported vigorous physical activity; OAA, Overall acceleration average; FAA, Fraction of accelerations > 425 milli-gravities;
