# Supplementary material for: Physical activities and risk of neurodegenerative diseases: A two-sample Mendelian randomization study
Source: Front Aging Neurosci. 2022 Sep 23;14:991140. doi: 10.3389/fnagi.2022.991140 (PMC9541335; doi:10.3389/fnagi.2022.991140)
Supplement: Supplementary file 2 [file Table_2.DOCX]

Additional file 2: Estimated association between physical activity phenotypes and Parkinson’s disease by using different mendelian randomization methods.

| PA phenotypes | MR methods | Number of SNPs | *p* | OR (95%CI) |
| --- | --- | --- | --- | --- |
| MVPA | IVW | 7 | 0.662 | 0.748 (0.203-2.754) |
|  | MR Egger | 7 | 0.735 | 0.240 (0-587.974) |
|  | Maximum likelihood | 7 | 0.602 | 0.742 (0.242-2.274) |
|  | Simple median | 7 | 0.365 | 0.449 (0.080-2.535) |
|  | Weighted median | 7 | 0.242 | 0.420 (0.098-1.799) |
| VPA | IVW | 5 | 0.876 | 0.831 (0.081-8.529) |
|  | MR Egger | 5 | 0.65 | 0.006 (0-2448141.536) |
|  | Maximum likelihood | 5 | 0.835 | 0.828 (0.141-4.857) |
|  | Simple median | 5 | 0.729 | 0.620 (0.041-9.278) |
|  | Weighted median | 5 | 0.988 | 0.982 (0.092-10.518) |
| OAA | IVW (RE) | 8 | 0.922 | 0.987 (0.766-1.273) |
|  | MR Egger | 8 | 0.313 | 0.502 (0.148-1.709) |
|  | Maximum likelihood | 8 | 0.690 | 0.984 (0.908-1.066) |
|  | Simple median | 8 | 0.221 | 1.061 (0.965-1.167) |
|  | Weighted median | 8 | 0.197 | 1.068 (0.966-1.181) |
| FAA | IVW (RE) | 8 | 0.259 | 0.394 (0.079-1.981) |
|  | MR Egger | 8 | 0.202 | 0 (0-994.765) |
|  | Maximum likelihood | 8 | 0.009^*^ | 0.378 (0.183-0.784) |
|  | Simple median | 8 | 0.404 | 0.682 (0.277-1.678) |
|  | Weighted median | 8 | 0.449 | 0.695 (0.271-1.781) |

^*^: *p* value less than 0.05 is considered statistically significant.

Abbreviations: PA, physical activity; MR, mendelian randomization; SNP, single nucleotide polymorphism; MVPA, Self-reported moderate-to-vigorous physical activity; VPA, Self-reported vigorous physical activity; OAA, Overall acceleration average; FAA, Fraction of accelerations > 425 milli-gravities; IVW, Inverse variance weighted; OR, odds ratio; CI, confidence interval.
