# Supplementary material for: Physical activities and risk of neurodegenerative diseases: A two-sample Mendelian randomization study
Source: Front Aging Neurosci. 2022 Sep 23;14:991140. doi: 10.3389/fnagi.2022.991140 (PMC9541335; doi:10.3389/fnagi.2022.991140)
Supplement: Supplementary file 3 [file Table_3.DOCX]

Additional file 3: Estimated association between physical activity phenotypes and Alzheimer’s disease by using different mendelian randomization methods.

| PA phenotypes | MR methods | Number of SNPs | *p* | OR (95%CI) |
| --- | --- | --- | --- | --- |
| MVPA | IVW | 6 | 0.632 | 1.247 (0.505-3.075) |
|  | MR Egger | 6 | 0.125 | 19.62 (0.906-401.027) |
|  | Maximum likelihood | 6 | 0.589 | 1.253 (0.552-2.846) |
|  | Simple median | 6 | 0.992 | 0.995 (0.337-2.932) |
|  | Weighted median | 6 | 0.651 | 1.288 (0.430-3.853) |
| VPA | IVW (RE) | 5 | 0.881 | 0.858 (0.116-6.367) |
|  | MR Egger | 5 | 0.796 | 0.072 (0-6265914.761) |
|  | Maximum likelihood | 5 | 0.829 | 0.855 (0.208-3.523) |
|  | Simple median | 5 | 0.587 | 0.566 (0.072-4.423) |
|  | Weighted median | 5 | 0.568 | 0.560 (0.076-4.106) |
| OAA | IVW (RE) | 6 | 0.506 | 1.031 (0.941-1.130) |
|  | MR Egger | 6 | 0.495 | 1.160(0.788-1.707) |
|  | Maximum likelihood | 6 | 0.258 | 1.034 (0.976-1.095) |
|  | Simple median | 6 | 0.269 | 1.045 (0.966-1.130) |
|  | Weighted median | 6 | 0.448 | 1.031 (0.953-1.116) |
| FAA | IVW (RE) | 7 | 0.773 | 0.905 (0.459-1.785) |
|  | MR Egger | 7 | 0.848 | 0.283 (0-57745.84) |
|  | Maximum likelihood | 7 | 0.671 | 0.899 (0.551-1.468) |
|  | Simple median | 7 | 0.336 | 1.475 (0.669-3.251) |
|  | Weighted median | 7 | 0.538 | 1.243 (0.621-2.487) |

Abbreviations: PA, physical activity; MR, mendelian randomization; SNP, single nucleotide polymorphism; MVPA, Self-reported moderate-to-vigorous physical activity; VPA, Self-reported vigorous physical activity; OAA, Overall acceleration average; FAA, Fraction of accelerations > 425 milli-gravities; IVW, Inverse variance weighted; OR, odds ratio; CI, confidence interval.
