# Supplementary material for: Physical activities and risk of neurodegenerative diseases: A two-sample Mendelian randomization study
Source: Front Aging Neurosci. 2022 Sep 23;14:991140. doi: 10.3389/fnagi.2022.991140 (PMC9541335; doi:10.3389/fnagi.2022.991140)
Supplement: Supplementary file 4 [file Table_4.DOCX]

Additional file 4: Sensitivity test of the mendelian randomization analysis between physical activity phenotypes and Parkinson’s disease and Alzheimer’s disease.

| Diseases | PA phenotypes | Methods | Heterogeneity test | | Horizontal pleiotropy | | MR-PRESSO | Steiger test |
| --- | --- | --- | --- | --- | --- | --- | --- | --- |
|  |  |  | Cochran’s Q | *p* | Egger_intercept | *p* | *p* of global test | *p* |
| PD | MVPA |  |  |  | 0.018 | 0.783 | 0.222 | 0^*^ |
|  |  | IVW | 8.350 | 0.214 |  |  |  |  |
|  |  | MR-Egger | 8.211 | 0.145 |  |  |  |  |
|  | VPA |  |  |  | 0.048 | 0.659 | 0.172 | 0^*^ |
|  |  | IVW | 7.077 | 0.132 |  |  |  |  |
|  |  | MR-Egger | 6.558 | 0.087 |  |  |  |  |
|  | OAA |  |  |  | 0.175 | 0.311 | <0.001^*^ | 0^*^ |
|  |  | IVW | 107.281 | 0^*^ |  |  |  |  |
|  |  | MR-Egger | 89.134 | 0^*^ |  |  |  |  |
|  | FAA |  |  |  | 0.444 | 0.222 | <0.001^*^ | 0.007^*^ |
|  |  | IVW | 43.493 | <0.001^*^ |  |  |  |  |
|  |  | MR-Egger | 33.208 | <0.001^*^ |  |  |  |  |
| AD | MVPA |  |  |  | -0.045 | 0.137 | 0.309 | <0.001^*^ |
|  |  | IVW | 6.185 | 0.289 |  |  |  |  |
|  |  | MR-Egger | 2.73 | 0.604 |  |  |  |  |
|  | VPA |  |  |  | 0.024 | 0.806 | 0.147 | <0.001^*^ |
|  |  | IVW | 8.179 | 0.085 |  |  |  |  |
|  |  | MR-Egger | 7.988 | 0.046^*^ |  |  |  |  |
|  | OAA |  |  |  | -0.034 | 0.573 | 0.063 | 0^*^ |
|  |  | IVW | 13.593 | 0.018 |  |  |  |  |
|  |  | MR-Egger | 12.423 | 0.014^*^ |  |  |  |  |
|  | FAA |  |  |  | 0.029 | 0.859 | 0.128 | 0^*^ |
|  |  | IVW | 12.279 | 0.056 |  |  |  |  |
|  |  | MR-Egger | 12.194 | 0.032^*^ |  |  |  |  |

^*^: *p* value less than 0.05 is considered statistically significant.

Abbreviations: PA, physical activity; MR, mendelian randomization; MVPA, Self-reported moderate-to-vigorous physical activity; VPA, Self-reported vigorous physical activity; OAA, Overall acceleration average; FAA, Fraction of accelerations > 425 milli-gravities; IVW, Inverse variance weighted; PD, Parkinson’s disease; AD, Alzheimer’s disease.
